# Direct mitral regurgitation quantification in hypertrophic cardiomyopathy using 4D flow CMR jet tracking: evaluation in comparison to conventional CMR

**DOI:** 10.1186/s12968-021-00828-y

**Published:** 2021-12-06

**Authors:** Aakash N. Gupta, Ryan Avery, Gilles Soulat, Bradley D. Allen, Jeremy D. Collins, Lubna Choudhury, Robert O. Bonow, James Carr, Michael Markl, Mohammed S. M. Elbaz

**Affiliations:** 1 Department of Radiology, Northwestern University, Feinberg School of Medicine, 737 N Michigan, Suite 1600, Chicago, IL 60611 USA; 2 Department of Radiology, Mayo Clinic, Rochester, MN 55902 USA; 3 Department of Medicine, Division of Cardiology, Northwestern University, Feinberg School of Medicine, Chicago, IL 60611 USA; 4 Department of Biomedical Engineering, Northwestern University, McCormick School of Engineering, Evanston, IL 60208 USA

**Keywords:** Hypertrophic cardiomyopathy, Mitral regurgitation, Quantification, 4D flow CMR

## Abstract

**Background:**

Quantitative evaluation of mitral regurgitation (MR) in hypertrophic cardiomyopathy (HCM) by cardiovascular magnetic resonance (CMR) relies on an indirect volumetric calculation. The aim of this study was to directly assess and quantify MR jets in patients with HCM using 4D flow CMR jet tracking in comparison to standard-of-care CMR indirect volumetric method.

**Methods:**

This retrospective study included patients with HCM undergoing 4D flow CMR. By the indirect volumetric method from CMR, MR volume was quantified as left ventricular stroke volume minus forward aortic volume. By 4D flow CMR direct jet tracking, multiplanar reformatted planes were positioned in the peak velocity of the MR jet during systole to calculate through-plane regurgitant flow. MR severity was collected for agreement analysis from a clinical echocardiograms performed within 1 month of CMR. Inter-method and inter-observer agreement were assessed by intraclass correlation coefficient (ICC), Bland–Altman analysis, and Cohen’s kappa.

**Results:**

Thirty-seven patients with HCM were included. Direct jet tracking demonstrated good inter-method agreement of MR volume compared to the indirect volumetric method (ICC = 0.80, p = 0.004) and fair agreement of MR severity (kappa = 0.27, p = 0.03). Direct jet tracking showed higher agreement with echocardiography (kappa = 0.35, p = 0.04) than indirect volumetric method (kappa = 0.16, p = 0.35). Inter-observer reproducibility of indirect volumetric method components revealed the lowest reproducibility in end-systolic volume (ICC = 0.69, p = 0.15). Indirect volumetric method showed good agreement of MR volume (ICC = 0.80, p = 0.003) and fair agreement of MR severity (kappa = 0.38, p < 0.001). Direct jet tracking demonstrated (1) excellent inter-observer reproducibility of MR volume (ICC = 0.97, p < 0.001) and MR severity (kappa = 0.84, p < 0.001) and (2) excellent intra-observer reproducibility of MR volume (ICC = 0.98, p < 0.001) and MR severity (kappa = 0.88, p < 0.001).

**Conclusions:**

Quantifying MR and assessing MR severity by indirect volumetric method in HCM patients has limited inter-observer reproducibility. 4D flow CMR jet tracking is a potential alternative technique to directly quantify and assess MR severity with excellent inter- and intra-observer reproducibility and higher agreement with echocardiography in this population.

**Supplementary Information:**

The online version contains supplementary material available at 10.1186/s12968-021-00828-y.

## Background

In obstructive hypertrophic cardiomyopathy (HCM), there is a direct link between left ventricular outflow tract (LVOT) obstruction and mitral regurgitation (MR) [1]. Specifically, elevated LVOT pressure gradients drive systolic anterior motion (SAM) of the anterior mitral valve leaflet [2]. Leaflet contact with the left ventricle (LV) septum and increased anterior motion of the anterior mitral leaflet leads to impaired leaflet coaptation and an eccentric posterolaterally directed MR jet [3]. Accurate evaluation of MR is critical since it is (i) a marker of LVOT disease severity that often improves with surgical treatment of the LVOT obstruction [4], (ii) a potential indicator of intrinsic valvular abnormalities that may warrant concomitant mitral valve surgery during septal myectomy [5], and (iii) a risk factor for left atrial dilatation and new onset atrial fibrillation [6].

Conventional cardiovascular magnetic resonance (CMR) methods indirectly quantify MR using a volumetric method: LV stroke volume (SV) minus forward aortic forward flow [7, 8]. LV SV is measured from planimetry-based LV volumetric contouring, and forward aortic flow is acquired from phase-contrast CMR (PC-CMR) [8]. However, recent HCM studies examining the indirect volumetric method have shown that LV SV is subject to significant variability based on ventricular contouring technique, and LVOT obstruction contributes to inaccuracy in aortic forward flow measurements [9, 10]. Thus, development and validation of CMR techniques to directly quantify the severity of MR in patients with HCM is warranted.

Recently, 4D flow CMR has emerged as a promising modality for direct quantitative assessment of valvular regurgitation [8, 11]. This direct quantification approach involves frame-by-frame tracking of regurgitant flow throughout the cardiac cycle and has demonstrated good agreement with standard-of-care CMR and reproducibility in various other pediatric [11–13] and adult [11, 14] populations. While this technique appears promising for regurgitant flow quantification, 4D flow CMR quantification of HCM-associated MR has not been compared to the indirect volumetric method. Additionally, SAM-mediated MR in HCM is typically late-systolic and eccentric, making it challenging to directly evaluate with existing modalities [7, 15]. Therefore, the aims of this study were to evaluate direct 4D flow CMR jet tracking for assessing severity of MR in HCM patients compared to the conventional CMR method (indirect volumetric method), with respect to inter-observer and intra-observer reproducibility, analysis time, and agreement with transthoracic echocardiography (TTE).

## Methods

### Study population

This is a retrospective study of adult patients with a diagnosis of HCM based on prior TTE who underwent a clinically indicated CMR with 4D flow CMR for HCM assessment. Patients were included if they had asymmetric-septal subtype of HCM and coverage of the mitral valve and left atrium on 4D flow CMR. Exclusion criteria included arrhythmias, other HCM phenotypes, prior valve repair or replacement, thoracic aortic aneurysm, congenital cardiac abnormalities, or incomplete LV short-axis stack or 2D PC-CMR of the aorta. Patients were identified by a retrospective chart review approved by the Institutional Review Board (IRB). Patients included in this study were previously reported in prior publications [16–18], none of which assessed direct MR quantification with 4D flow CMR.

### CMR

Imaging was performed on 1.5T or 3T CMR systems (Avanto, Aera, Skyra, Siemens Healthineers, Erlangen, Germany). Electrocardiogram (ECG)-gated time-resolved balanced steady-state free precession (bSSFP) cine imaging in two-chamber, three-chamber, four-chamber, LVOT, and LV short-axis stack was performed. Aortic 2D PC-CMR at the sinotubular junction with through-plane velocity encoding was acquired. Gadolinium-based contrast (Gadavist, Bayer Pharmaceuticals, Berlin, Germany) was intravenously administered in all patients. 4D flow CMR was acquired as the last sequence of the exam. Two board-certified cardiovascular radiologists (J.D.C. and J.C.) measured LV maximal wall thickness (MWT) on end-diastolic LV short-axis bSSFP cines and assessed for SAM of the mitral valve on three-chamber bSSFP cine [3].

### 4D flow CMR

Time-resolved 3D, phase-contrast CMR with three-directional velocity encoding (4D flow CMR) with prospective ECG- and respiratory-gating was acquired in a three-chamber orientation to evaluate the left atrium, LV, and LVOT. Acquisition time ranged between 8 and 15 min, depending on heart rate and respiratory navigator efficiency. Acquisition parameters included spatial resolution (2.1–3.3) × (2.1–3.3) × (2.4–4.0) mm^3^, temporal resolution 36.8–39.2 ms, velocity encoding (VENC) 150–250 cm/s, echo time 2.2–2.5 ms, flip angle 15°, field of view (225–400) × (255–420) mm^2^, and slab thickness 65–176 mm. Data were pre-processed to correct for Maxwell terms, eddy currents, and velocity aliasing using cvi42 (version 5.9, Circle Cardiovascular Imaging, Calgary, Alberta, Canada).

### Transthoracic echocardiography

MR severity was retrospectively collected from clinically interpreted TTEs obtained with standard views. In brief, per clinical guidelines, MR was graded by integrating qualitative, semi-quantitative, and quantitative parameters including effective regurgitant orifice area, proximal isovelocity surface area method, and regurgitant volume/fraction [7]. Patients with TTE within 1 month of CMR were included for comparison with CMR-based quantification. The rationale behind this 1-month inclusion criterion was to allow for a consistent comparison, mitigating potential pathophysiological temporal variability in MR while balancing the statistical power to test the agreement. However, for completeness and transparency, agreement to all available TTE data within 1 week, 2 weeks, 1 month, 3 months, and 6 months of CMR was also tested and reported in Appendix 1.

### MR quantification with indirect volumetric method

All analyses was performed using cvi42 (Circle Cardiovascular Imaging). A certified cardiovascular radiologist with 9 years of CMR experience (G.S.) calculated routine LV cardiac function parameters by contouring the bSSFP short-axis stack of the LV. Trabeculae and papillary muscles were excluded from the LV blood pool volume using semi-automated algorithms. Forward flow in the aorta was quantified from 2D PC-CMR of the aorta with background offset correction [8]. MR volume was calculated as LV SV minus forward aortic flow [7].

### MR quantification with 4D flow CMR jet tracking

**Fig. 1:**
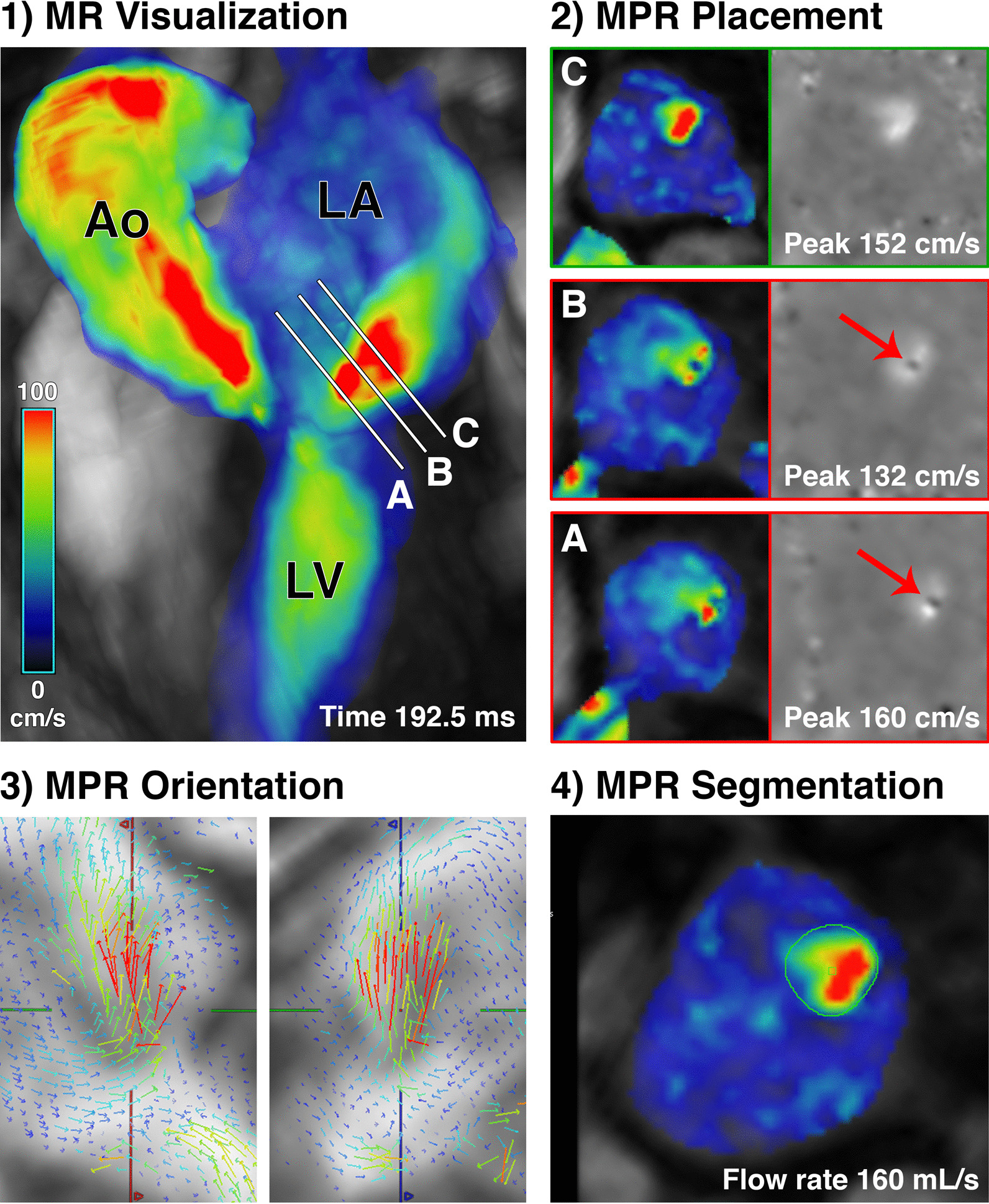
Multiplanar reformatted (MPR) plane analysis for direct jet tracking. **1** Velocity color-coded 4D flow data of the left atrium (LA), left ventricle (LV), and aorta (Ao) in mid-systole show an MR jet in the left atrium (LA). To illustrate proper MPR plane placement, three candidate planes A, B, and C (white lines) are placed in the MR jet with increasing distance from the mitral valve. **2** Color-coded and corresponding grayscale through-plane velocity images are shown for planes A–C. Only plane C is suitable for analysis because it contains the maximum velocity without aliasing. Planes A and B are unsuitable due to aliasing within the MR jet (red arrows). **3** For plane C, vector glyphs with double-oblique views are used to orient the plane perpendicular to the MR jet flow. **4** The through-plane velocity of the MR jet is carefully segmented to calculate regurgitant flow rate. This analysis is repeated for each timeframe with MR

**Fig. 2:**
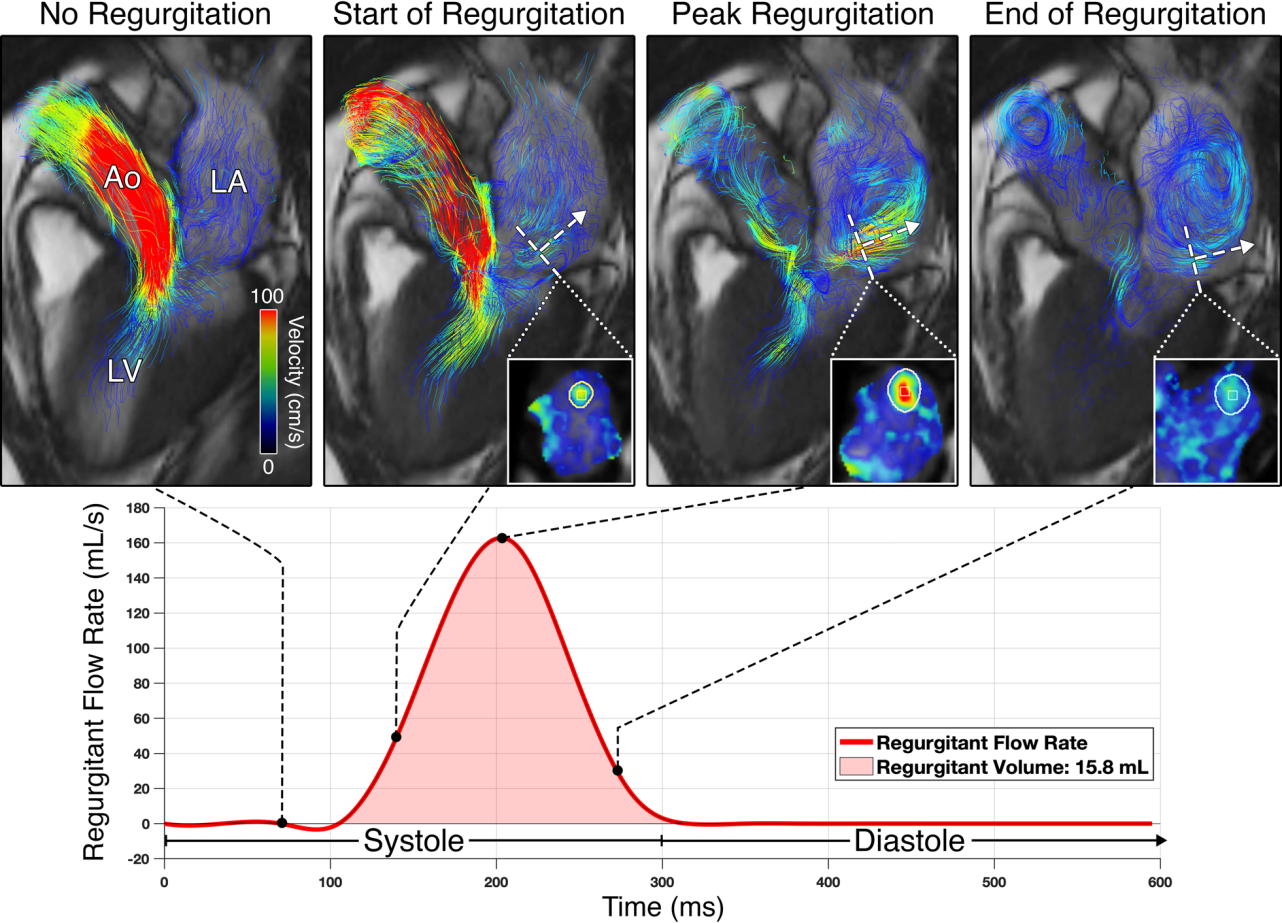
4D flow CMR jet tracking analysis for direct MR quantification. LA blood flow is visualized throughout systole to look for MR. For each timeframe with MR, an MVPR reformatted plane (dashed line) is positioned at the peak velocity of the MR jet, oriented orthogonal (dashed arrow) to the jet, and contoured to capture the cross-section of the jet and calculate the regurgitant flow rate (Fig. 1). The resulting regurgitant flow rate curve is spline-interpolated and integrated over the MR jet period to calculate MR volume. The MPR planes dynamically track and adapt to the jet direction over systole. In this example, a 52 year-old male with hypertrophic cardiomyopathy (HCM) (maximal wall thickness (MWT) 2.4 cm) and a typical mid-to-late systolic posterolateral MR jet is depicted with an MR volume of 16 mL, consistent with mild MR (see Additional file 1: Video S1)

Figures 1 and 2 illustrates the methodology of direct MR quantification using 4D flow CMR jet tracking in a patient with HCM. Methodology is based on prior work by Calkoen et al. in patients with corrected atrioventricular septal defects [12]. In summary, left atrial blood flow was inspected over systole using both 3D color-coded velocity images and pathlines to identify time frames of the cardiac cycle in which MR was present. For each time frame with MR, a separate multiplanar reformatted (MPR) plane was positioned at the peak velocity within the MR jet and oriented perpendicularly to the jet direction, followed by manual contouring of the jet cross-section on the through-plane velocity MPR plane (cvi42, v5.9, Circle Cardiovascular Imaging). MPR planes were positioned further in the jet to avoid regions of aliasing. This technique provided a regurgitant flow rate at each systolic time frame with identified MR. Subsequently, the time-resolved regurgitant flow-rate curve was spline-interpolated and integrated to calculate the MR volume for each patient.

For multiple MR jets, each jet was independently tracked, and the regurgitant volumes for each jet were summed to calculate the total MR volume. If no timeframes with a regurgitant jet were identified, regurgitant volume was zero. Jet-tracking analysis was performed by an observer with 2 years of CMR experience (A.N.G.) who was blinded to MR volume measurements by indirect volumetric method. Intra-observer analysis was repeated in a blinded fashion 1 year after the initial analysis.

### Inter-observer reproducibility

A board-certified cardiovascular radiologist (R.A.) with 8 years of CMR experience repeated indirect volumetric method and direct 4D flow CMR jet tracking in a blinded manner and randomized order. Analyses were repeated for all patients with a 3–4 week interval between indirect volumetric method and 4D flow CMR jet tracking. Analysis times for indirect volumetric method and 4D flow jet tracking were recorded for comparison.

### Assessment of sources of measurement variability

To identify sources of measurement variability, inter-observer reproducibility was assessed separately for each of the components involved in the indirect volumetric method computation: LV end-diastolic volume (EDV), end-systolic volume (ESV), SV, and aortic forward flow. Similarly, for 4D flow direct jet tracking, the reproducibility of identification of the MR start frame, end frame, and MR duration were assessed.

### Classification of MR severity

MR volume measurements for both indirect volumetric method and 4D flow CMR jet tracking methods were classified by severity of MR, defined using MR volume: none (< 10 mL), mild (10–30 mL), moderate (30–60 mL), and severe (≥ 60 mL) [7].

### Statistical analysis

Given the relatively limited sample size, continuous values are reported as median [interquartile range (IQR)], and IQR is reported as [25%, 75%]. Categorical data are reported as percentage. To assess the agreement between MR volume measurements between the indirect volumetric method and direct 4D flow jet tracking, measurements from both observers were averaged for each method and compared with intraclass correlation coefficient (ICC) and Bland–Altman analysis. Similarly, inter-observer and intra-observer measurements were tested with ICC (two-way random, single measures, absolute agreement) and Bland–Altman analysis. ICC values were interpreted as follows: moderate (0.50–0.70), good (0.71–0.85), strong (0.86–0.95), and excellent (0.96–1.00). Bland–Altman limits of agreement (LOA) were calculated as mean ± 1.96 * standard deviation (SD). Cohen’s kappa was calculated to assess agreement of MR severity. Kappa values were interpreted as the following: poor (0), slight (0.01–0.20), fair (0.21–0.40), moderate (0.41–0.60), substantial (0.61–0.80), and excellent (0.81–1.00) agreement [19]. Analysis times between methods were compared by paired Wilcoxon signed-rank test. A p-value < 0.05 was considered statistically significant. Statistical analysis was performed using Matlab (version R2018b, MathWorks, Natick, Massachusetts, USA).

## Results

### Study cohort

The final study cohort consisted of 37 patients with HCM (52.1 [46.1, 64.5] years, 15 female) with a median LV ejection fraction (LVEF) of 63.5% (IQR, [60.4, 67.0] %) (Table 1). SAM was present in 23 patients (62.2%). Median LV mass was 178.9 g (IQR [137.9, 195.6] g) and MWT was 2.0 cm (IQR [1.7, 2.3] cm).

**Table 1:** Patient characteristics, cardiac function parameters, and HCM assessment parameters

	HCM (n = 37)
Patient characteristics	
Age (years)	52.1 [46.1, 64.5]
Male	22 (59.5%)
Cardiac function	
LV end-diastolic volume (mL)	134 [122, 152]
LV end-systolic volume (mL)	41 [35, 49]
LV stroke volume (mL)	95 [78, 112]
Ejection fraction (%)	70.6 [64.8, 73.2]
Heart rate (bpm)	68.0 [59.1, 76.4]
Aortic forward flow (mL)	70 [61, 83]
Forward cardiac output (L/min)	4.5 [3.7, 6.0]
HCM assessment	
LV mass (g)	179 [138, 196]
Max wall thickness (cm)	2.0 [1.7, 2.3]
SAM present (%)	23 (62.2%)
MR volume	
Indirect volumetric method (mL)	20 [12, 38]
Direct 4D flow jet tracking (mL)	13 [4, 23]

Values are listed as median [IQR] or count (frequency)

*LV* left ventricle, *SAM* systolic anterior motion, *LVOT* left ventricular outflow tract, *MR* mitral regurgitation

### Comparison of CMR-based quantification methods

**Fig. 3:**
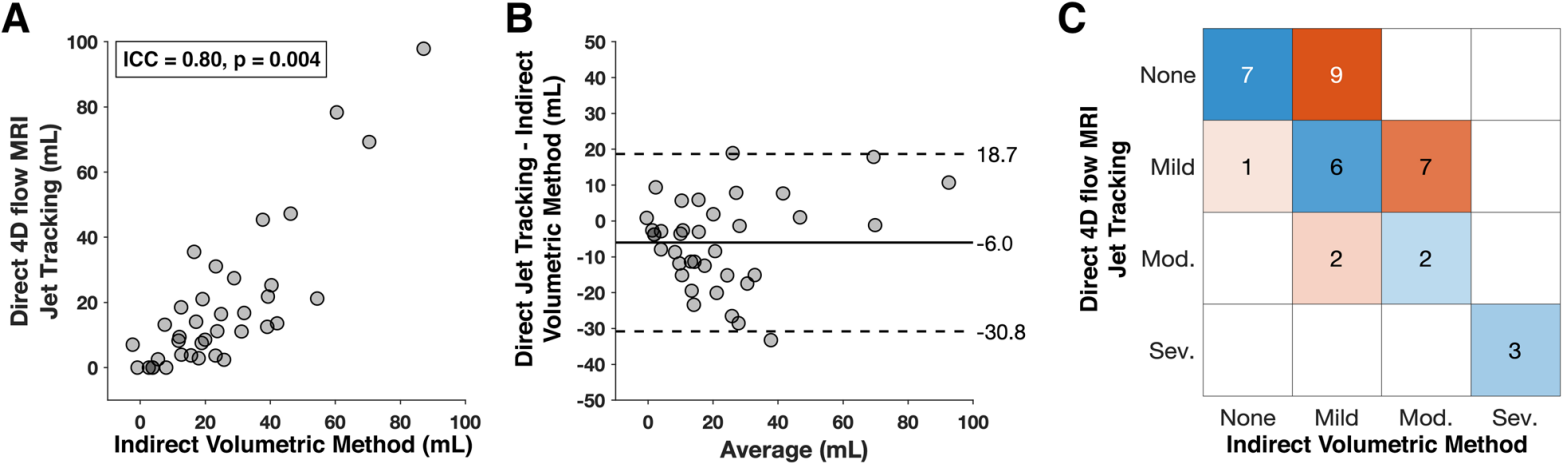
Comparison of MR quantification by direct 4D flow CMR jet tracking with indirect volumetric method (LV stroke volume—aortic forward flow). **A** Correlation of MR volumes with intraclass coefficient (ICC), **B** Bland–Altman agreement shown with bias and LOA, and **C** agreement of MR severity classification, in which cases with agreement (blue) and cases with disagreement (orange) are shaded proportional to total number of cases

Five patients (13.5%) did not show a regurgitant jet on 4D flow CMR using either color-coded velocity images or pathline visualization. Median MR volume by indirect volumetric method for these 5 patients was 3 mL (IQR [2, 14] mL). Two MR jets were identified in one patient with the jets measuring 10 mL and 7 mL. MR volume showed good agreement (ICC = 0.80, p = 0.004) between indirect volumetric method and direct 4D flow CMR jet tracking (Fig. 3A). Compared to indirect volumetric method, Bland–Altman analysis revealed an underestimation of 6 mL (LOA: [− 31, 19] mL) by 4D flow CMR jet tracking (Fig. 3B). Agreement between both methods on MR severity classification was fair (kappa = 0.27, p = 0.03; Fig. 3C). Agreement was seen in 18 patients (48.6%), disagreement by one MR severity grade was seen in 19 patients (51.4%), and zero cases disagreed by more than one severity grade (Fig. 3C). Analysis time was significantly faster for the 4D flow CMR jet tracking method compared to the conventional indirect volumetric method (7.0 [3.0, 9.3] min vs. 8.0 [6.0, 12.3] min, p = 0.03).

### Comparison with transthoracic echocardiography

**Fig. 4:**
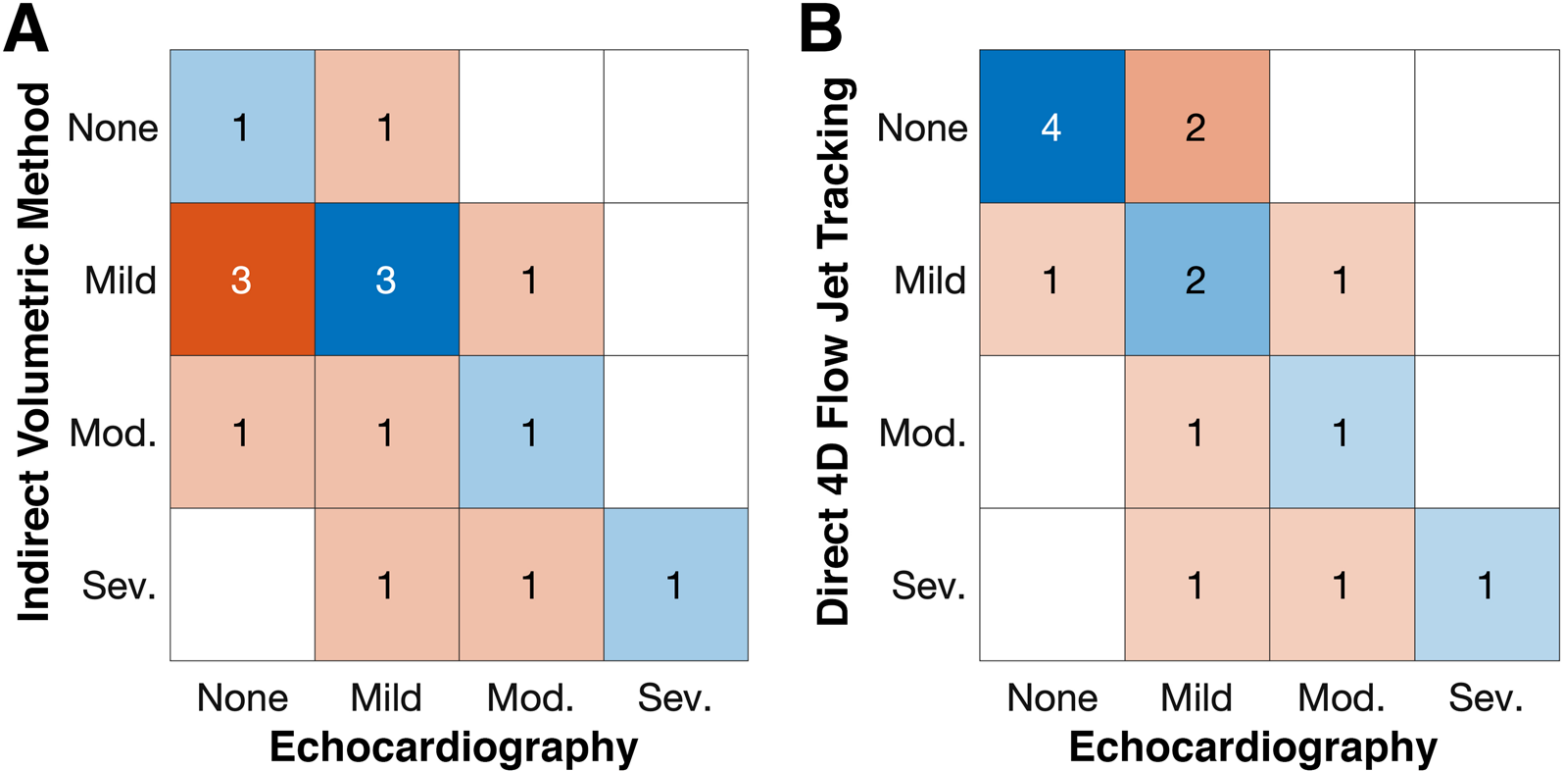
MR severity agreement between transthoracic echocardiography and CMR-based methods including **A** indirect volumetric method and **B** direct 4D flow CMR jet tracking

Fifteen patients had an TTE within 1 month of CMR. The indirect volumetric method demonstrated only slight agreement (kappa = 0.16, p = 0.35; Fig. 4A); whereas direct 4D flow CMR jet tracking demonstrated fair agreement (kappa = 0.35, p = 0.04; Fig. 4B) with TTE. Notably, direct jet tracking demonstrated a higher agreement than the indirect volumetric method when using TTE data within 1 week, 2 weeks, 1 month, 3 months, and 6 months of CMR (Appendix 1).

### Sources of measurement variability

**Fig. 5:**
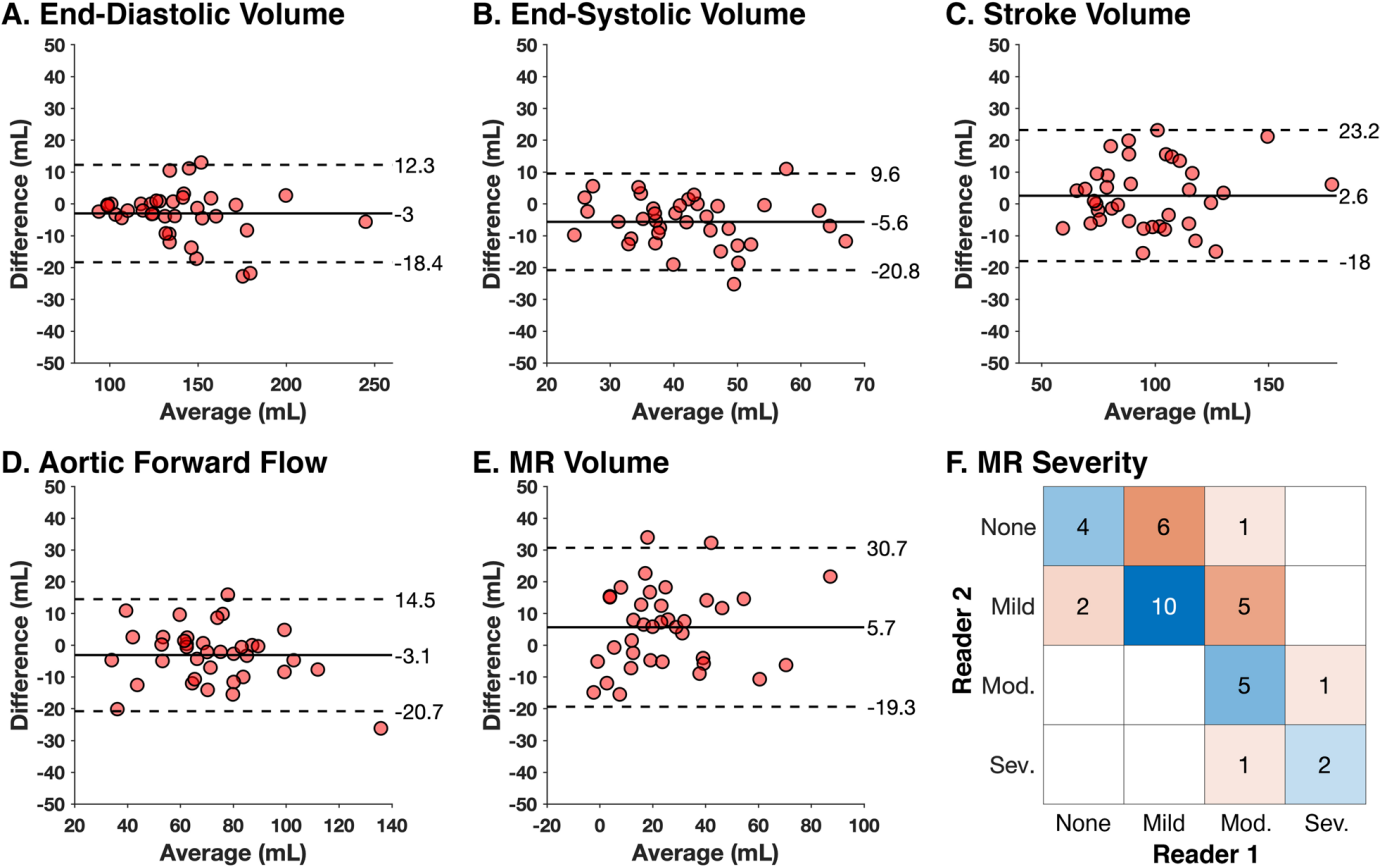
Inter-observer analysis for indirect volumetric quantification measurements (**A**–**D**), MR volume (**E**), and MR severity (**F**). Bland–Altman plots are shown for **A**–**E** with bias and limits of agreement indicated on the right of each plot. In **F**, inter-observer agreement (blue) and disagreement (orange) for classification of MR severity classification is shown with boxes shaded proportionally to the number of cases

Table 2 summarizes the results of inter-observer analysis for the measurements used in both methods. EDV (ICC = 0.97, p < 0.001; Fig. 5A) showed excellent reproducibility (bias: − 3 mL, LOA: [− 18, 12] mL). However, ESV showed the lowest reproducibility with wide limits of agreement (bias: − 6 mL, LOA: [− 21, 10] mL) and lowest ICC (ICC = 0.69, p = 0.15; Fig. 5B). LVSV (ICC = 0.91, p < 0.001; Fig. 5C) and aortic forward flow (ICC = 0.91, p < 0.001; Fig. 5D) demonstrated strong agreement.

**Table 2:** Inter-observer analysis for indirect volumetric method measurements and direct 4D flow CMR measurements

	Bland–Altman	Intraclass coefficient		Cohen’s kappa^a^	
	Bias [LOA]	ICC	p-value	Kappa	p-value
Indirect volumetric method					
LV end-diastolic volume (mL)	− 3 [− 18, 12]	0.97	< 0.001*	–	–
LV end-systolic volume (mL)	− 6 [− 21, 10]	0.69	0.15	–	–
LV stroke volume (mL)	3 [− 18, 23]	0.91	< 0.001*	–	–
Aortic forward flow (mL)	− 3 [− 21, 15]	0.91	< 0.001*	–	–
MR volume (mL)	6 [− 19, 31]	0.80	0.003*	0.38	< 0.001*
Direct 4D flow CMR jet tracking					
MR start (timeframe #)	0 [− 2, 2]	0.90	< 0.001*	–	–
MR end (timeframe #)	0 [− 1, 1]	1.00	< 0.001*	–	–
MR jet duration (no. of timeframes)	0 [− 1, 1]	0.99	< 0.001*	–	–
MR volume (mL)	− 2 [− 12, 8]	0.97	< 0.001*	0.84	< 0.001*

*LOA* limits of agreement, *ICC* intraclass coefficient, *MR* mitral regurgitation

Asterisk (*) denotes significant p-value < 0.05

^a^Cohen’s kappa was calculated for severity of MR (none, mild, moderate, severe), which was derived from MR volume

For 4D flow jet tracking, identification of start frame of MR had strong inter-observer agreement (ICC = 0.90, p < 0.001). The identified end frame of MR (ICC = 1.0, p < 0.001) and total MR duration (ICC = 0.99, p < 0.001) both had excellent inter-observer agreement.

### Inter-observer analysis for indirect volumetric method

Figure 5 and Table 2 summarize the results of inter-observer analysis. MR volume showed good agreement (bias = 6 mL, LOA = [− 19, 31] mL; ICC = 0.80, p = 0.003; Fig. 5E) and fair agreement in classification of MR severity (kappa = 0.38, p < 0.001; Fig. 5F). Additionally, to compare with prior studies, inter-observer results using LV volumes indexed to body surface area (BSA), calculated by Mosteller method, are provided in Appendix 2.

### Inter- and intra-observer analyses for direct 4D flow CMR jet tracking

**Fig. 6:**
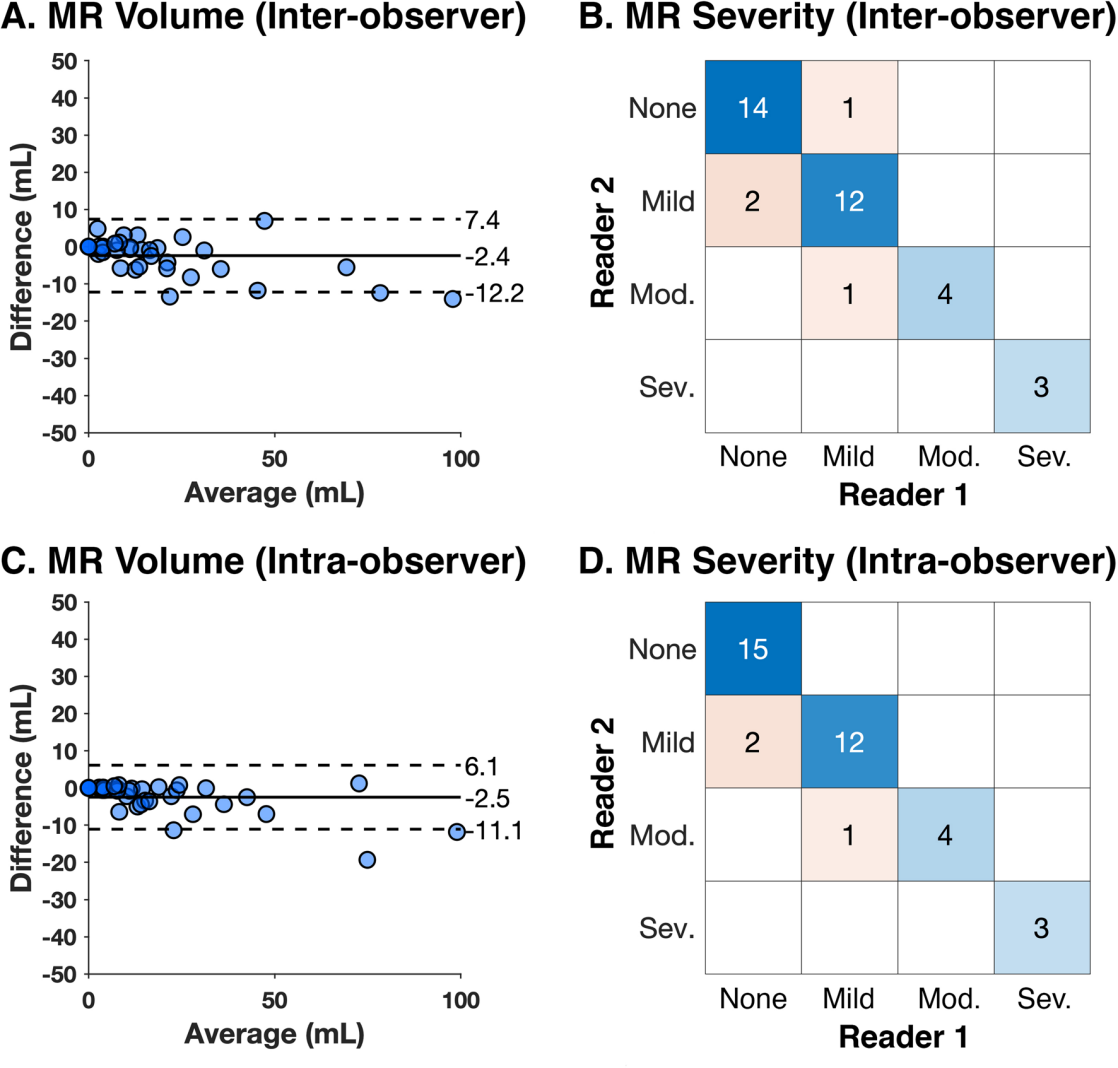
Inter-observer analysis (**A**, **B**) and intra-observer analysis (**C**, **D**) for MR volume and MR severity measured by direct 4D flow CMR jet tracking

Figure 6 and Table 2 shows the results of inter-observer analysis. Of note, both observers agreed on the absence of MR in 5 patients but disagreed in one patient (MR volume 5 mL versus 0 mL). Direct measurement of MR volume displayed excellent reproducibility (bias = − 2 mL, LOA = [− 12, 8] mL; ICC = 0.97, p < 0.001; Fig. 6A). Classification of MR severity also demonstrated excellent agreement (kappa = 0.84, p < 0.001; Fig. 6B).

Intra-observer analysis demonstrated excellent reproducibility (bias = − 2.5 [− 11.1, 6.1] mL; ICC = 0.98, p < 0.001; Fig. 6C) and excellent agreement of MR severity (kappa = 0.88, p < 0.001; Fig. 6D).

## Discussion

In this study, we sought to directly quantify MR in patients with HCM using 4D flow CMR jet tracking and to evaluate this technique in comparison to standard-of-care indirect volumetric method from CMR. The key findings are as follows: (1) Direct 4D flow CMR jet tracking demonstrated good agreement in MR quantification with standard-of-care indirect volumetric method, but with notable variability. (2) Direct jet tracking had higher agreement with TTE than the indirect volumetric method. (3) Inter-observer analysis of the indirect volumetric method components demonstrated that most parameters were reproducible, but variability increased as measurements were combined. (4) For MR quantification, inter-observer analysis demonstrated that: (i) indirect volumetric method showed lower reproducibility with only good agreement in MR volume and fair agreement in classification of MR severity, and (ii) direct 4D flow CMR jet tracking showed excellent agreement of MR volume and excellent classification of MR severity.

### MR quantification in HCM

Assessment of MR severity is an important component of the evaluation of HCM. Yu et al*.* illustrated that MR severity reflects LVOT pressure gradients and is reduced when septal myectomy reduces LVOT obstruction [4]. Given that SAM-mediated MR is typically posteriorly directed, an anterior or central MR jet has served as a potential indicator of MR secondary to concomitant mitral valve disease [5, 20]. Additionally, chronic MR and LV diastolic dysfunction lead to increased left atrial pressure with compensatory dilatation and remodeling [6]. Left atrial dilation is a significant predictor of the development of new-onset atrial fibrillation, which increases the risk of thromboembolism by eight-fold in HCM [21, 22].

CMR-based MR quantification using the indirect volumetric approach has its limitations. LV SV and aortic forward flow are computed from different sequences (bSSFP and PC-CMR) which have their own potential inter-observer variability and physiologic variability due to heart rate fluctuations between acquisitions. An important limitation of the indirect volumetric method is the potential error propagation arising from subtracting EDV, ESV, and aortic forward volume from each other, which may increase the relative error of the final MR volume (Fig. 5) [7, 23].

In HCM, measurements of aortic flow and LV SV are subject to additional challenges. Spiewak et al*.* identified that complex aortic flow patterns arising from LVOT obstruction led to underestimation of aortic flow on PC-CMR in reference to main pulmonary arterial flow [9]. Inclusion of hypertrophied trabeculations and papillary muscles within the LV blood pool contouring may also overestimate LV SV [9, 10, 24, 25]. Both findings, individually and together, would likely lead to overestimation of MR volume. In this study, we excluded trabeculae and papillary muscles from the LV blood pool. The median LV SV was 95 mL (IQR, 78–112 mL). Notably, this LV SV is comparable to two other studies in HCM cohorts that excluded trabeculae and papillary muscle: (1) Spiewak et al. found a median of 90 mL (IQR, 78–105 mL) [9] and (2) Han et al. found a mean of 98 mL (standard deviation 25 mL) [10]. This may further indicate the robustness of our analysis pipeline and results. Likewise, the inter-observer reproducibility reported for EDV in this study (bias − 2 mL/m^2^, LOA [− 9, 6] mL/m^2^, Appendix 2) and SV (bias 1 mL/m^2^, LOA [− 9, 12] mL/m^2^, Appendix 2) are comparable to Han et al. [10]. Here, we found that ESV was the least reproducible parameter, possibly due to hypertrophied muscles obscuring the endocardial border in end-systole. By the indirect volumetric method, inter-observer agreement of MR severity disagreed in 43.2% (16/37) of all cases (Fig. 5F). These findings support the need for careful consideration when using indirect volumetric methods to quantify MR in HCM.

### 4D flow CMR for direct MR quantification

Direct quantification of regurgitation is particularly beneficial in complex cases of multivalvular disease or intracardiac shunting and is made possible with 4D flow CMR [8]. Retrospective valve tracking was first introduced by Westenberg et al. and utilizes MPR planes defined by the valve annulus position on bSSFP images to quantify regurgitant and transvalvular flow on 4D flow CMR data [26]. Valve tracking has demonstrated high internal consistency of net flow across cardiac valves [11, 27] and external agreement with CMR volumetric method [12] and TTE [28]. However, valve tracking requires additional bSSFP orthogonal views for each valve of interest to track the respective annulus. When mapping the valve annulus to the 4D flow data, differences in breath holding techniques may result in misalignment necessitating careful spatial registration [8]. Additionally, eccentric jets that do not pass perpendicularly through the valve may pose an additional challenge [29].

The jet tracking analysis in this study is similar to prior studies in that MPR planes are manually placed at a supravalvular position directly within the jet using only 4D flow CMR data [13, 14]. Measuring at a supravalvular position may have the benefit of minimizing turbulence-related signal voids located at the valve level while also allowing for dynamic adaptation to eccentric and time-varying jets. Our methodology differs in that we identify the peak velocity within the jet on 4D flow data as a flow-based landmark to position MPR planes consistently. Jet tracking analysis only utilizes 4D flow CMR data and does not require additional bSSFP scans. In circumventing additional acquisitions and spatial registration steps, jet tracking requires users to navigate 4D flow CMR data, identify presence of MR, and define timepoints containing MR. Here, we found high inter-observer reproducibility in identification of timepoints with MR (ICC = 0.99), quantification of MR volume (ICC = 0.97), and classification of MR severity (kappa = 0.84). When comparing direct jet tracking to indirect volumetric method, we found a comparable but larger underestimation and limits of agreement (− 6.0 [− 30.8, 18.7] mL) compared to a pediatric population of corrected atrioventricular septal defects reported by Calkoen et al. (− 5 [− 20, 12] mL) [12]. This may be, in part, due to aforementioned overestimations of MR volume with the indirect volumetric method specific to HCM (e.g. LVOT obstruction and LV papillary muscle segmentation technique) [9, 10].

In assessing agreement with TTE, our analysis was limited to scans within 1 month of CMR to balance data availability, statistical power, and potential temporal pathophysiologic MR variability. Discrepancy may arise from physiologic fluctuations in volume status and blood pressure, alterations in medications between scans, or disease progression. However, in comparison to the indirect volumetric method, our results demonstrated that the direct jet tracking-based MR quantification had consistently higher agreement in MR severity with TTE quantification irrespective of the inclusion timeframe for TTE scans which may further support our results (Appendix 1). These findings could be, in part, due to the similarity between the two techniques in using direct interrogation of MR jet properties in evaluating MR severity. Similar to the direct jet tracking method, TTE also depends on direct assessment of MR jet properties including jet direction, regurgitant area, and peak velocity. Whereas the indirect method does not directly probe such jet properties.

From continuous-wave Doppler TTE studies, we expect that the peak velocity (approximately 4–6 m/s) of an MR jet occurs at the regurgitant orifice [7]. However, in 4D flow CMR, the captured peak velocity within the jet is often lower in velocity (dependent on venc) and located at a supravalvular position in the left atrium (Figs. 1 and 2). This is likely the case for a few reasons: (1) lower spatial and temporal resolution of 4D flow CMR will lead to intra-voxel averaging of high velocities with lower velocities and will lower the recorded peak velocity; (2) flow displacement effect from high velocity spins within the MR jet traveling between phase-encoding and frequency readout [30]; and (3) turbulence-associated signal loss at the valvular level from mitral valve apparatus motion and the high-velocity MR jet itself [15].

### Limitations

There are several limitations to our study. The absence of a true reference standard for MR quantification makes validation of new techniques and determination of accuracy challenging. Here, we primarily validated our results against CMR as a clinical standard-of-care reference and focused on assessing agreement, reproducibility, and analysis time. Agreement with TTE was limited by varying time between TTE and CMR, data availability, and multiple readers. Future studies comparing same day TTE and CMR may be necessary to confirm the initial findings of this study. Next, prospective ECG-gated 4D flow CMR was used which has incomplete temporal coverage of end-diastole. Retrospectively ECG-gated acquisitions would be necessary to assess flow consistency across all four valves as well as aortic and pulmonic regurgitation [11, 27]. However, jet tracking analysis focuses on quantification of MR and not on diastolic mitral inflow. In addition, follow-up scans with 4D flow CMR were not available in this retrospective cohort to assess scan–rescan variability or variability with acquisition resolutions, but potential impact of such variabilities on direct jet tracking should be evaluated in the future.

## Conclusions

In patients with HCM, direct 4D flow CMR jet tracking demonstrated an overall good agreement, but with notable variability, against standard-of-care indirect volumetric method for quantification of MR. ESV was the main source of inter-observer variability in the conventional indirect MR quantification. Compared to conventional CMR, direct 4D flow CMR jet tracking demonstrated higher reproducibility of MR volume and severity as well as improved agreement with MR severity determined by TTE. Analysis time was faster using the direct jet tracking method versus the indirect method. These results highlight the clinical challenges with utilizing the indirect volumetric method in HCM and support 4D flow CMR jet tracking as a potential alternative technique with high reproducibility to directly quantify MR in HCM patients.

### Supplementary Information

**Additional file 1: Video S1.** 4D flow CMR streamline visualization of mitral regurgitation in a patient with HCM.

## Data Availability

The datasets used and/or analyzed during the current study are available from the corresponding author on reasonable request.

## Appendices

### Appendix 1: Comparison between CMR and echocardiogram MR severity

See Table 3.

**Table 3:** Agreement of MR severity using clinical echocardiograms available within increasing timeframes from the date of CMR

	Indirect volumetric method		Direct 4D flow CMR method	
	Kappa	p-value	Kappa	p-value
Echocardiogram				
1 week (n = 6)	0.08	0.80	0.54	0.07
2 weeks (n = 7)	0.15	0.58	0.60	0.03
1 month (n = 15)	0.16	0.35	0.35	0.04
3 months (n = 27)	0.17	0.20	0.37	0.04
6 months (n = 30)	0.15	0.21	0.25	0.04

### Appendix 2: Inter-observer analysis using volumes indexed to BSA

See Table 4.

**Table 4:** Inter-observer analysis of LV volumes indexed to BSA

	Bland–Altman	Intraclass coefficient	
	bias [LOA]	ICC	p-value
LV end-diastolic volume indexed (mL/m^2^)	− 1.5 [− 9.3, 6.3]	0.95	< 0.001*
LV end-systolic volume indexed (mL/m^2^)	− 2.8 [− 10.6, 4.9]	0.67	0.18
LV stroke volume indexed (mL/m^2^)	1.4 [− 9.1, 11.9]	0.88	< 0.001*
Aortic forward flow indexed (mL/m^2^)	− 1.5 [− 9.9, 6.9]	0.89	< 0.001*
MR volume indexed (mL/m^2^)	2.9 [− 9.5, 15.3]	0.81	0.002*

Asterisk (*) denotes significant p-value < 0.05

## Abbreviations

Ao: Aorta
BSA: Body surface area
bSSFP: Balanced steady-state free precession
CMR: Cardiovascular magnetic resonance
ECG: Electrocardiogram
EDV: End-diastolic volume
ESV: End-systolic volume
HCM: Hypertrophic cardiomyopathy
LV: Left ventricle/left ventricular
LVEF: Left ventricular ejection fraction
LVOT: Left ventricular outflow tract
MPR: Multiplanar reformatted
MR: Mitral regurgitation
PC: Phase-contrast
SAM: Systolic anterior motion
SV: Stroke volume
TTE: Transthoracic echocardiography
VENC: Velocity encoding
